# Genome-wide DNA methylation and transcriptome analyses reveal genes involved in immune responses of pig peripheral blood mononuclear cells to poly I:C

**DOI:** 10.1038/s41598-017-10648-9

**Published:** 2017-08-29

**Authors:** Haifei Wang, Jiying Wang, Chao Ning, Xianrui Zheng, Jinlian Fu, Aiguo Wang, Qin Zhang, Jian-Feng Liu

**Affiliations:** 1 Key Laboratory of Animal Genetics, Breeding and Reproduction, Ministry of Agriculture, College of Animal Science and Technology, China Agricultural University, Beijing, 100193 China; 2 Shandong Key Laboratory of Animal Disease Control and Breeding, Institute of Animal Science and Veterinary Medicine, Shandong Academy of Agricultural Sciences, Jinan, 250100 China

## Abstract

DNA methylation changes play essential roles in regulating the activities of genes involved in immune responses. Understanding of variable DNA methylation linked to immune responses may contribute to identifying biologically promising epigenetic markers for pathogenesis of diseases. Here, we generated genome-wide DNA methylation and transcriptomic profiles of six pairs of polyinosinic-polycytidylic acid-treated pig peripheral blood mononuclear cell (PBMC) samples and corresponding controls using methylated DNA immunoprecipitation sequencing and RNA sequencing. Comparative methylome analyses identified 5,827 differentially methylated regions and 615 genes showing differential expression between the two groups. Integrative analyses revealed inverse associations between DNA methylation around transcriptional start site and gene expression levels. Furthermore, 70 differentially methylated and expressed genes were identified such as *TNFRSF9*, *IDO1* and *EBI3*. Functional annotation revealed the enriched categories including positive regulation of immune system process and regulation of leukocyte activation. These findings demonstrated DNA methylation changes occurring in immune responses of PBMCs to poly I:C stimulation and a subset of genes potentially regulated by DNA methylation in the immune responses. The PBMC DNA methylome provides an epigenetic overview of this physiological system in response to viral infection, and we expect it to constitute a valuable resource for future epigenetic epidemiology studies in pigs.

## Introduction

Epigenetic alterations are a regular and natural occurrence that can result in heritable changes in gene expression without changing the underlying DNA sequence while being influenced by genetic changes and environmental cues^1^. It has received intense attention due to their functions in regulating chromatin structure and gene expression, and their potential use as a biomarker for disease status^2^. In particular, DNA methylation is considered as the most stable and experimentally accessible biomarker candidate among different types of epigenetic marks. Characterization of DNA methylome is of importance for understanding the functional mechanisms of DNA methylation in human disease studies^3^. DNA methylation signatures have been found to be predictive of a series of diseases, including melanoma^2^, Wilms tumor^4^ and major depressive disorder^5^, which will be of great value in both clinical diagnostics and therapeutics. Furthermore, DNA methylation changes can regulate the transcriptional profiles of innate and memory lymphocytes and thus shape their function. It has been reported that DNA methylation alterations in activating molecules such as *KSR1* and *NCF4* are associated with human natural killer cell activation^6^. DNA methylation can direct transcription of the genes encoding cytokines, effector molecules and their receptors such as *IFNγ*, *IL2* and *CCR6* in memory T cells^7–9^. The survival of CD4+ memory T cells has been found to be affected by DNA methylation status of the cell death-related gene *NOXA* ^10^. Dynamic changes of demethylation and remethylation occur at the programmed cell death-related gene *PDCD1* which encodes a key regulator of cell proliferation and exhaustion during naive to effector to functional memory CD8+ T cell differentiation post viral infection^11^.

Peripheral blood mononuclear cells (PBMCs) are clinically relevant cells that display a variety of both innate and adaptive immune functions and have well-established roles in surveillance for infectious threats. PBMCs methylome at single base-pair resolution demonstrated it to be rich in biological information in humans^12^. Recently it has also been shown that global methylation of PBMCs has the potential to provide disease-specific epigenetic signatures^13, 14^. Moreover, DNA methylation was found to be strongly associated with PBMC responsitivity to Toll-like receptor (TLR) ligands^15^. The TLR3 agonist polyinosinic-polycytidylic acid (poly I:C), an artificial surrogate for double-stranded RNA (dsRNA) virus, has been extensively used to simulate viral infection^16^. As such kind of external pathogenic stimuli can conduce to epigenetic changes, understanding of the variable DNA methylation linked to immune and inflammatory responses of PBMCs may contribute to identifying biologically promising epigenetic markers for pathogenesis of viral diseases.

As one of the important biomedical models for human diseases, methylation patterns of diverse pig tissues have been reported^17, 18^. Epigenetic analysis of porcine dendritic cells treated by lipopolysaccharide highlighted the involvement of epigenetic mechanisms in innate immune response^19^. In addition, pig PBMCs has been frequently used as an experimental model to investigate immune responses to vaccination, bacterial and viral infection^20–22^. As an appealing biomedical model with high similarities to humans in anatomy, genetics and pathophysiology, the immune system of pigs not only has a full set of innate and adaptive immune effectors, but also closely resembles humans for >80% of analyzed immune parameters, which enables the effective uses of pigs in modeling human immune responses and microbial infectious diseases^23, 24^. Epigenetic regulatory mechanisms are of particular importance to establish a proper pattern of gene expression in immune cells in response to internal or external environmental variations^25^. However, changes in epigenetic profile of pig PBMCs induced by pathogenic stimuli remain largely unknown so far. Studies of epigenetic responses involved in immune reactions of pig PBMCs upon pathogens may shed light on the epigenetic dynamics during both animal and human immunological responses.

In this study, using the pig as a model we established an *in vitro* model system to analyze changes in methylome profiling of PBMCs in response to poly I:C stimulation and explore potential roles of DNA methylation in this process. The methylated DNA immunoprecipitation sequencing (MeDIP-seq) was utilized to provide broad coverage of methylomic landscape of poly I:C-treated samples and controls. Integrative analysis of genome-wide DNA methylation and gene expression data was further performed to identify associations between epigenomic and transcriptomic responses of PBMCs to viral infection. The comprehensive analysis of PBMC methylome herein will advance our understanding of epigenetic contributions to immune responses to viral infection, and aid in the identification of potential epigenetic biomarkers associated with responsiveness to viruses.

## Results

### Genome-wide DNA methylation pattern

To probe the DNA methylation patterns of PBMCs that occur shortly after poly I:C infection, we performed comparative methylome analysis on six pairs of poly I:C-treated and control PBMC samples. After 24 hours of incubation, the samples were subjected to MeDIP-seq to generate a total of approximately 1.89 × 10^9^ paired-end reads with the length of 100 bp from 12 samples, of which 1.11 × 10^9^ high quality reads were mapped to the pig genome (Sscrofa10.2), resulting in approximately 9.25 × 10^7^ mapped reads per sample (Table S1). Based on the high-quality mapped reads, we conducted CpG coverage analysis at genome wide by using the MEDIPS package^26^. For each sample, on average 82.96% (range, 80.19% to 84.77%) of the approximately 28.0 million CpG sites in the pig genome were covered by at least one read (Table S2). Furthermore, saturation analysis indicated that we have sufficient reads to generate a reproducible genome-wide methylation profile (Figure S1).

To examine methylation changes at a genome-wide level, we quantified methylation levels on each chromosome using a 10 kb sliding window to smooth the distribution. The results showed that most chromosomal regions were covered by MeDIP-Seq reads, while the densities were distinct among these chromosomes (Figure S2). Further analysis indicated that regions with the densities of 51 to 55 CpGs/kb and CpG_o/e_ ratio close to 0.60 tend to exhibit a higher methylation level (Figures S3 and S4). In addition, the methylation level in subtelomeric regions (7 Mb downstream of the telomere) in most chromosomes was significantly increased compared with non-subtelomeric regions (Student’s t test, *P* < 0.01, Figure S5). This result supported the idea that subtelomeric sequences can be methylated in mammals^27^, and coincided with the highly methylated state of subtelomeric regions previously identified in mouse embryonic stem cells^28^ and in porcine adipose and muscle tissues^17^.

**Figure 1:**
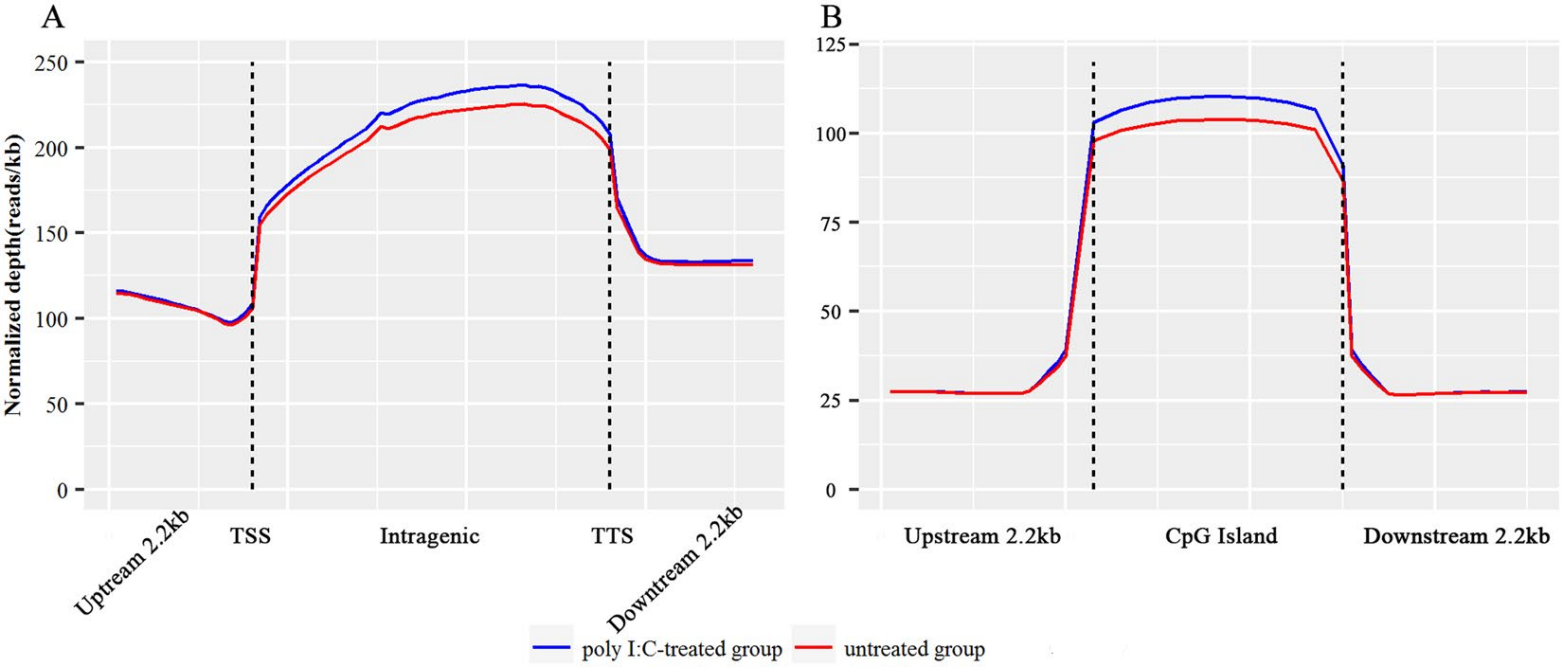
Distribution of MeDIP-seq reads in genomic features. (**A**) Methylation level in the gene region. The gene region consists of 2.2 kb upstream of the TSS and downstream of the TTS regions, and gene body from TSS to TTS. In gene body, each gene was divided into 50 equal windows, and the 2.2 kb regions upstream of TSS and downstream of TTS were divided into 20 non-overlapping windows. The average normalized depth was calculated for each window. TSS: transcription start site; TTS: transcription termination site. (**B**) Methylation level in CpG islands and CpG island shores. The CpG islands were split into 20 equal windows, and the CpG islands shores were split into 20 non-overlapping windows, and the average normalized read depth was calculated for each window. The Y-axis represents the average of normalized read depth for each window.

Next, we performed comprehensive analysis of the distribution of DNA methylation in the 2.2 kb region upstream of the transcription start site (TSS), gene body, and 2.2 kb region downstream of the transcription termination site (TTS). We observed a clear trend for DNA methylation levels to decrease sharply at TSS and to increase dramatically towards the gene bodies (Fig. 1A). This is concordant with the methylation pattern identified in human peripheral blood mononuclear cells^12^ and embryonic stem cells^29^, bovine muscle tissue^30^, as well as chicken live and muscle tissues^31^, which indicated that the methylation pattern of CpG hypermethylation within gene body regions is probably conserved among species. Moreover, the methylation level of gene bodies in the poly I:C-treated group was obviously higher (*P* < 0.01) than in the control group (Fig. 1A), which may be attributed to the potential associations between gene body methylation and expression of active genes.

In methylome studies, CpG islands (CGIs) are a notable genomic element of interest because of the role in controlling gene expression^32^. We therefore analyzed the methylation status of CGIs and CGI shores, and observed that the DNA methylation level increased sharply towards the CGIs (Fig. 1B). Of note, the poly I:C-treated group had relatively higher methylation levels in CGIs than the untreated group (*P* < 0.01), which suggests CGIs may contain regulatory sites of gene expression in responses of PBMCs to poly I:C stimulation. The DNA methylation changes in these genomic features presumably reflected the ability of pathogens to trigger epigenetic responses of cells involved in the immune system^33^.

### Identification of differentially methylated regions

**Figure 2:**
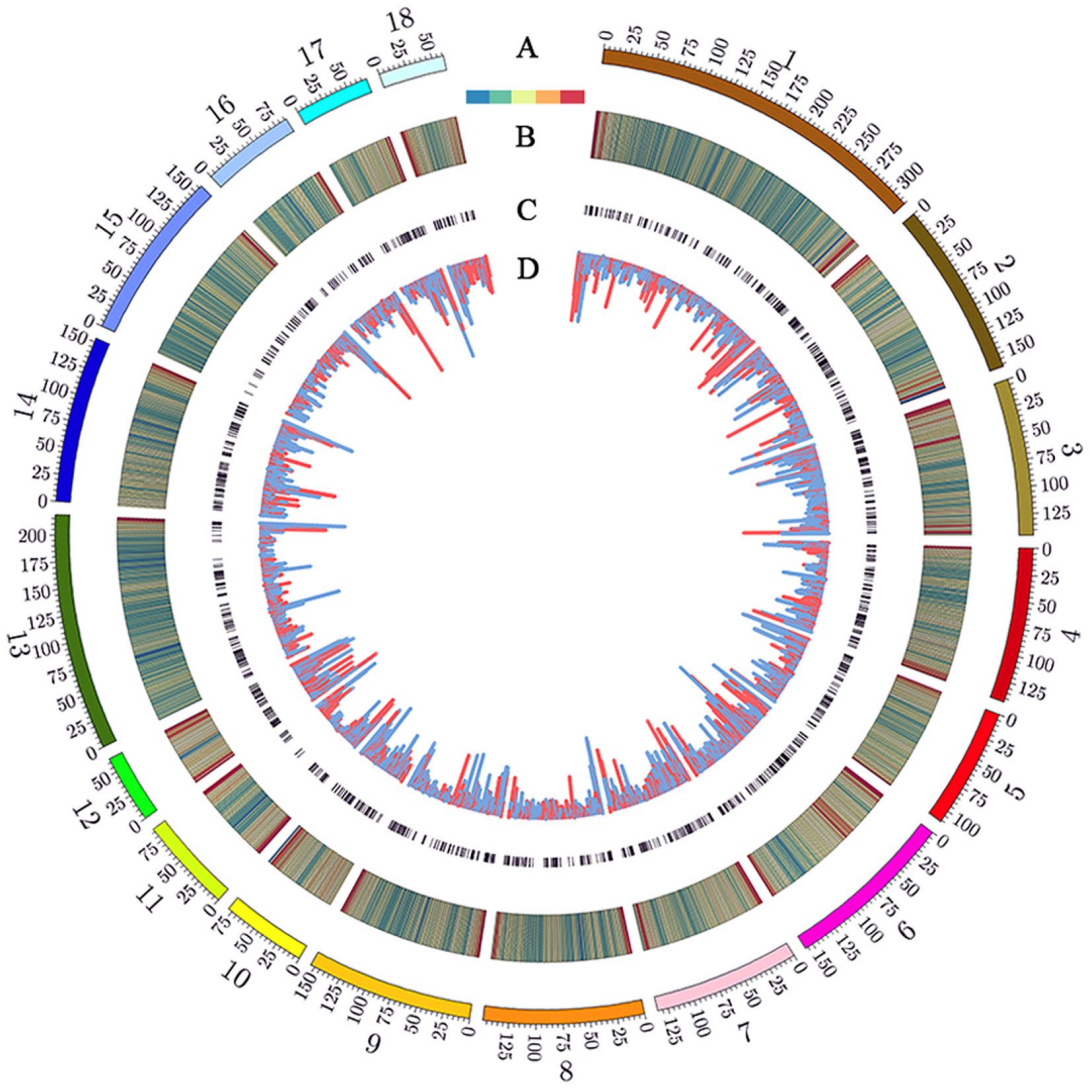
Genome-wide patterns of differentially methylated regions. (**A**) Circle diagram of pig autosomal chromosomes; (**B**) CpG_o/e_ ratio at 1 Mb bins; (**C**) 1,890 differentially methylated genes; (**D**) distribution of DMRs (the height of each histogram bar represents number of CpGs in the DMRs), of which the hyper- and hypomethylated DMRs are shown in orange and blue, respectively.

To identify the DMRs between poly I:C-treated and control PBMC samples, we calculated reads density in 1 kb sliding windows across the whole genome and performed pairwise comparisons between the two groups. As neighboring CpG sites are usually coordinately methylated up to 1 kb^34^, DMRs were defined as 1 kb windows with significant difference in normalized read counts between the two groups. We identified 5,827 DMRs, of which 2,322 (39.8%) were hypermethylated and 3,505 (60.2%) hypomethylated in the poly I:C-treated group. A circle diagram of DMRs along chromosomes revealed that distributions of DMRs tend to be enriched in genomic regions with higher CpG_o/e_ ratio (~0.32) than the genomic mean value (~0.22, Fig. 2, Table S3). To confirm the MeDIP-seq results, we carried out bisulfite sequencing PCR on four DMR regions in the poly I:C-treated and control PBMC samples used in MeDIP-seq. Results indicated that the MeDIP-seq data were highly correlated with bisulfite sequencing PCR data, confirming the reliability of the MeDIP-seq data (Pearson’s r = 0.92, *P* = 2.82e-13, Figure S6).

**Figure 3:**
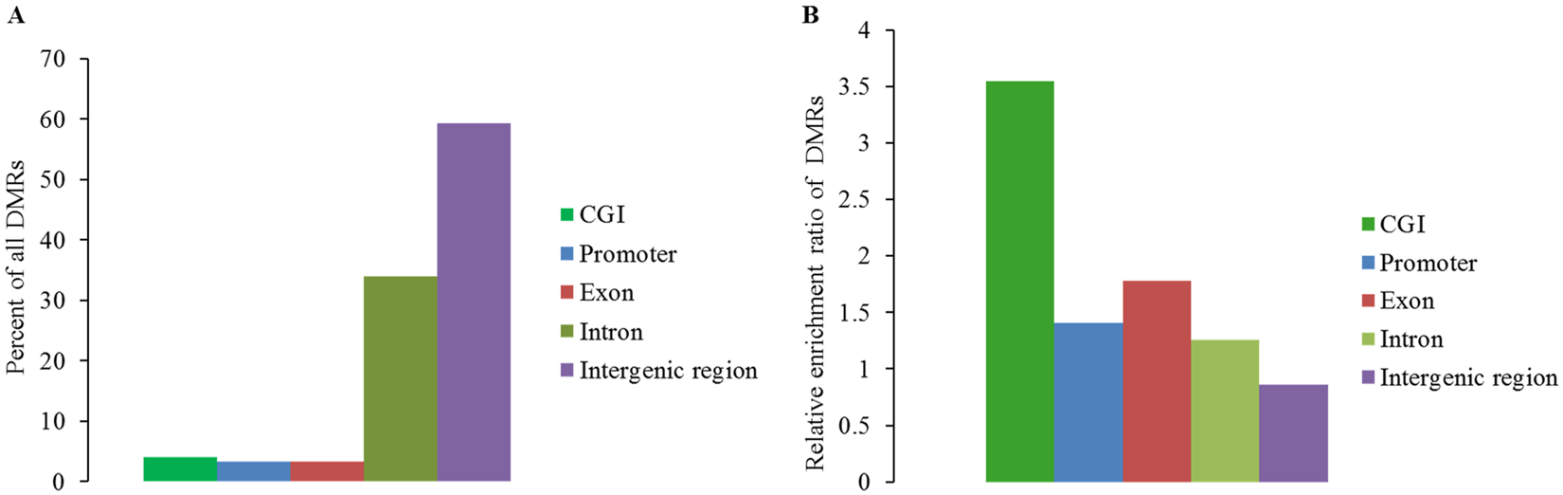
Distribution of DMRs in different genomic features. (**A**) Assignment of DMRs to CGI, promoter, exon, intron and intergenic region. (**B**) Relative enrichment ratio of DMRs. Enrichment ratio was calculated by the proportions of DMRs mapped to each genomic feature in total DMRs divided by the relative size of corresponding genomic features. Genomic coordinates for these feature types were obtained from the UCSC pig reference genome (Sscrofa 10.2), with the exception of promoters, which were defined as −2,200 bp to +500 bp of the transcription start site.

**Figure 4:**
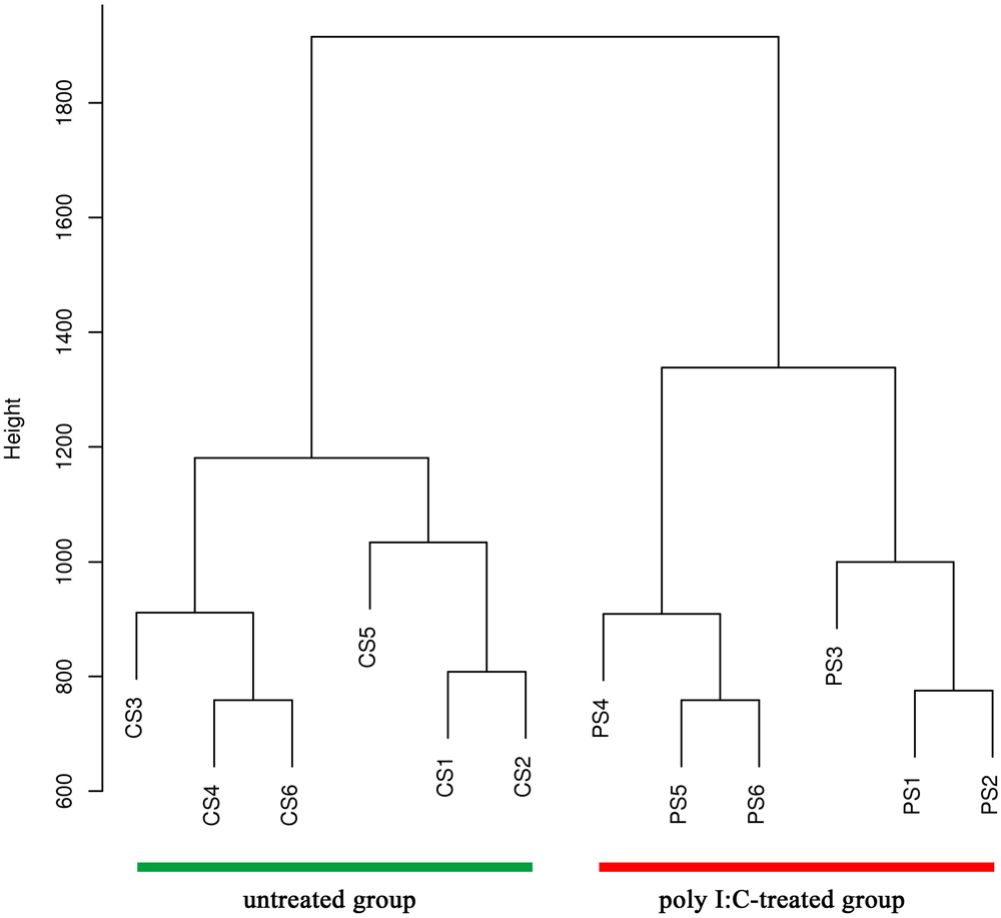
Hierarchical clustering of poly I:C-treated and untreated PBMC samples using methylation patterns present at DMRs. Clustering was performed using the hclust function in R.

We subsequently mapped all DMRs to their nearest genomic features and found that methylation alterations were unevenly distributed across the genome. The majority of the 5,827 DMRs were identified in intergenic and intronic regions (Fig. 3A), which constitute a large proportion of the genome. However, relative enrichment analysis revealed that the DMRs displayed higher enrichment in CGIs, exon and promoter regions (Fig. 3B). The unsupervised clustering analysis for all samples using DMRs clearly distinguished the two groups (Fig. 4), which reflects the potential to classify immune disease with DNA methylation changes in PBMCs. To predict the potential functional significance of the 5,827 DMRs identified in the poly I:C-treated group, we determined which regions overlapped with promoters and genes. We observed that the DMRs were associated with 1,896 genes, among which 142 genes were differentially methylated only in promoter, 1,663 only in gene bodies, and 91 in both promoters and gene bodies (Tables S4 and S5). Some of the genes with immune functions have been well studied, for example, Nod like C3 (*NLRC3*) that attenuates Toll-like receptor signaling via modification of the signaling adaptor TRAF6 and transcription factor NF-κB^35^, forkhead box P1 (*FOXP1*) that participates in the transcriptional regulatory network of B lymphopoiesis^36^.

As the differences in the gender and breed of individuals used in this study, we also revealed the potential DNA methylation differences derived from gender and pig breed differences. A total of 6,152 DMRs (2,114 hypomethylated and 4,038 hypermethylated) were detected between the different pig breeds, and 2,208 DMRs (1,414 hypomethylated and 794 hypermethylated) were identified between the individuals with different gender (Tables S6 and S7).

### Transcriptional response of PBMCs to poly I:C infection

**Figure 5:**
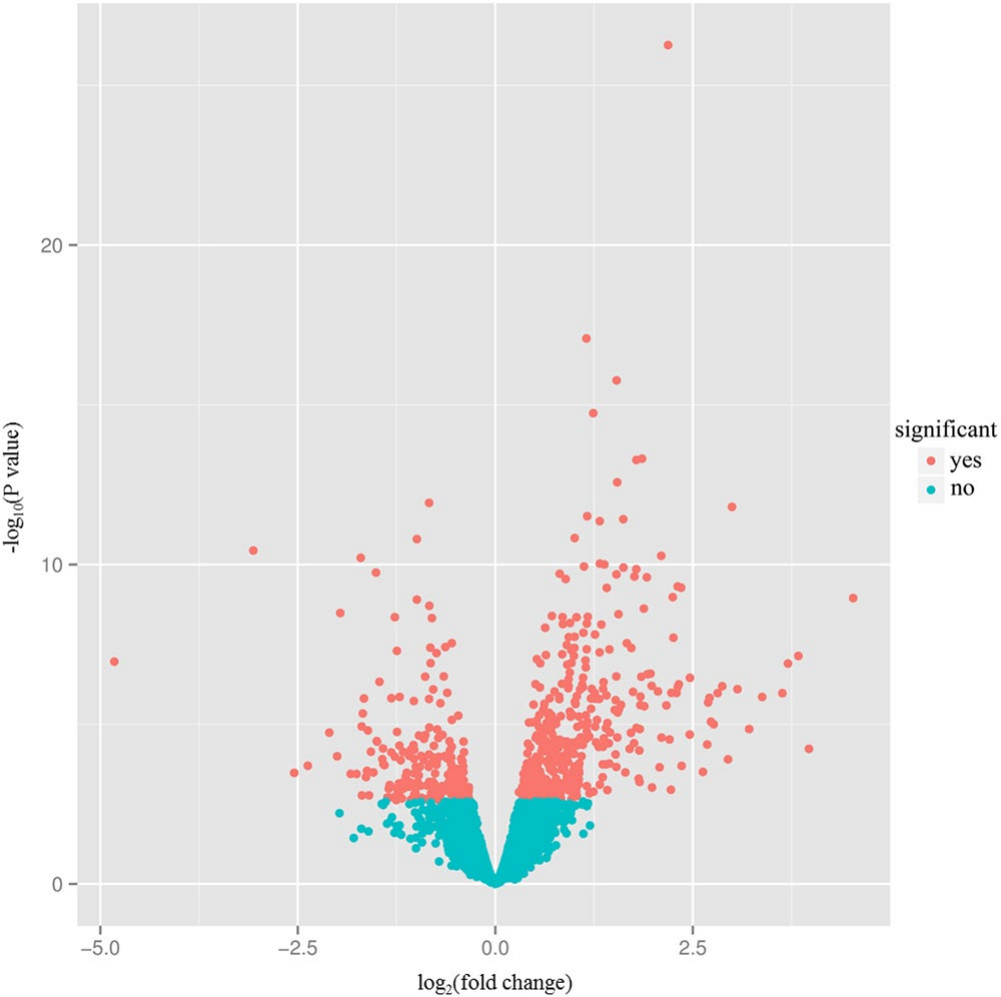
Visualization of significantly differentially expressed genes between the poly I:C-treated and untreated PBMC samples using a volcano plot. The horizontal line indicates the log2 fold change value, and the vertical line denotes the negative log10 p-values from the differential expression test. The red dots represent the differentially expressed genes (FDR < 0.05), and the cyan dots represent the genes that were not expressed differentially between the two groups.

Using RNA-seq to measure global gene expression in paired poly I:C-treated/untreated PBMC samples, we identified 615 genes that were significantly differentially expressed (FDR < 0.05, Table S8), with 424 genes up-regulated and 191 down-regulated in response to poly I:C infection (Fig. 5). This genome-wide expression analysis provided a comprehensive portrait of the immune responses to poly I:C infection at the transcriptional level. The unsupervised clustering analysis of samples by mRNA expression profile did not clearly separate the two groups (Figure S7), which is consistent with previous reports^37^. To investigate the biological significance of the differentially expressed genes, we undertook functional annotation analysis using DAVID bioinformatics resources. A subset of immune/defense response-related categories were significantly clustered, such as ‘response to wounding’ (GO: 0009611, 81 genes, FDR = 2.94E-28), ‘inflammatory response’ (GO: 0006954, 60 genes, FDR = 2.54E-24), and ‘defense response’ (GO: 0006952, 71 genes, FDR = 4.91E-17). Further pathway analysis revealed that the genes were significantly enriched in six disease resistance-related pathways. The cytokine-cytokine receptor interaction pathway (46 genes, FDR = 1.41E-11) was the most representative pathway, followed by graft-versus-host disease (14 genes, FDR = 1.66E-05), type I diabetes mellitus (13 genes, FDR = 4.16E-04), allograft rejection (12 genes, FDR = 6.13E-04), asthma (10 genes, FDR = 5.98E-03), and chemokine signaling pathway (24 genes, FDR = 2.87E-02) (Table S9).

To validate the RNA-seq analysis results, we selected ten differentially expressed genes that with differing expression patterns, including *CCL2*, *IL-6*, *IL-10*, *PPARG*, *TNFRSF9*, *TNF-α*, *AIG1*, *ADAM28*, *FCRL3*, *NCF4*, and *PACSIN1* to detect their expression patterns using qRT-PCR. The expression pattern of all the genes tested matched that was observed in the RNA-seq analysis results, with a correlation coefficient of 0.94 (*P* = 1.72E-05), confirming the high reliability and accuracy of RNA-seq data in this study (Figure S8).

### Integrative analyses of DNA methylation and gene expression data

**Figure 6:**
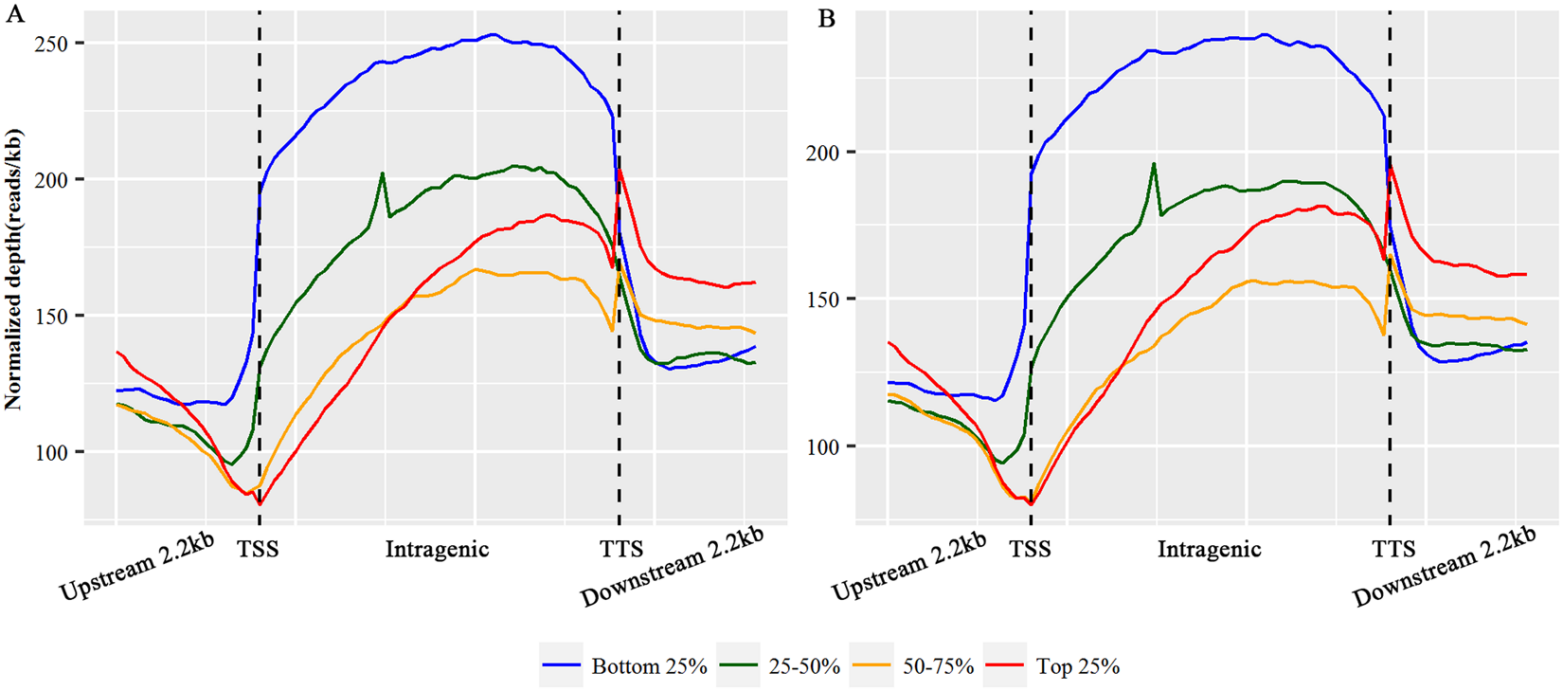
Relationship between DNA methylation and gene expression levels. The methylation level in the gene regions was estimated by the normalized read depth. In gene body, each gene was divided into 50 equal windows, and the average normalized depth was calculated for each window. The 2.2 kb regions upstream of TSS and downstream of TTS were divided into 20 non-overlapping windows, and the average normalized depth was calculated for each window. Genes were divided into four groups based on the ranking of average expression levels. Each line denotes the DNA methylation level of different gene expression groups. (**A**) Poly I:C-treated group; (**B**) untreated group.

To explore the effects of DNA methylation on the regulation of gene expression in PBMCs, we investigated the correlations between gene’s methylation level and expression level by integrating DNA methylation data with gene expression data generated from the same cohort of PBMC samples. We divided the genes into four groups according to the mean expression levels (Fig. 6). The silently expressed genes that were not detected in the two groups were removed. Then we compared the DNA methylation levels in and around gene bodies among the four expression levels in the two groups (Fig. 6). The top 25% highly expressed genes had the lowest methylation levels around the TSS of genes and groups of genes with increasingly lower expression levels exhibited increasingly higher methylation levels. This means that the highly methylated genes were expressed at low levels, and vice versa. A similar negative correlation between DNA methylation and gene expression levels surrounding TSS has been previously found in human peripheral blood monocytes^12^, bovine muscle tissue^30^, and chicken live and muscle tissues^31^. Genome-wide analysis of the correlation between methylation and transcription revealed significant negative correlations (r = −0.387, *P* = 2.2e-16). To test the relationship between promoter methylation and CpG frequency, we classified all promoters into three types based on the CpG representation, i.e., low CpG (LCP, n = 5,577), intermediate CpG (ICP, n = 8,599) and high CpG content promoters (HCP, n = 8,635) (Figure S9A). We observed an increase of methylation levels in ICPs compared with HCPs and LCPs (Figure S9B), which coincided with previous findings that ICPs show a high frequency of methylation and thus are prone to regulation by DNA methylation^38^.

Differential gene expression analysis in stimulated PBMCs *in vitro* highlighted the roles of differentially expressed genes in response to pathogenic stimuli^21, 39, 40^. To detect whether the differentially expressed genes identified in this study were associated with differential DNA methylation, we performed integrative analyses of genes identified in the DNA methylation and gene expression analyses. The results showed that among the list of differentially expressed genes, 70 genes were discovered to be differentially methylated between the two groups (Table S10), which was larger than expected to occur by chance (chi-square test, *P = *1.89E-03). The analysis allowed the identification of a subset of antiviral response-related genes including *TNFRSF9* that is expressed on activated T and natural killer cells^41^, *IDO1* that can suppress viral replication^42^ and *EBI3* that induces generation of regulatory T cells^43^, of which the genomic locations and degrees of the methylation and expression differences are shown in Figure S10. Gene ontology analysis revealed significant enrichment for categories including ‘positive regulation of immune system process’ (GO: 0002684, 6 genes, *P* = 2.75E-03) and ‘pattern binding’ (GO: 0001871, 5 genes, *P* = 2.87E-03), and ‘lysosome’ (GO: 0005764, 5 genes, *P* = 1.19E-02) (Table S11).

## Discussion

Through a genome-wide comparative methylome analysis of pig PBMCs, we revealed the associations of epigenetic alterations with the responses of PBMCs to immune stimulation by the viral pathogen. We characterized differentially methylated regions that reflect the epigenetic changes potentially induced by poly I:C stimulation. Further integrated analysis of MeDIP-seq and RNA-seq data allowed the identification of immune response-related genes that may be driven by DNA methylation. The PBMC DNA methylome profiling provides an epigenetic overview of this physiological system in response to *ex vivo* stimulation, and we expected it to constitute a subset of resources for future epigenomic studies.

It is known that pathogens can affect diverse epigenetic factors such as DNA methylation, histone modifications, noncoding RNAs^44^. Our methylome analysis of poly I:C-treated PBMCs demonstrated distinct distribution patterns of differential DNA methylation among genomic features. In agreement with previous studies showing different distributions of methylation changes in genomic features^45, 46^, these suggest the potential of DNA methylation profile in reflecting the unique epigenetic information of immune responses and diseases. Consistent with the clear stratification of immune disease by DNA methylation signature^37^, our work further substantiated the general validity of the associations between DNA methylation and immune responses by unsupervised clustering analysis using DMRs. Some factors such as differences associated with sex and breed could impact on the DNA methylation^17^, and our findings also displayed sex and breed related DNA methylation differences which may result in that the individuals of Landrace and Dapulian could not be distinguished in the untreated group. In addition, individuals from the two breeds were separated in the poly I:C-treated group. Previous studies have shown the discrepancies in the ability of the two pig breeds in resistance to pathogenic stimulation^47^ and the close link between DNA methylation status and functions of immune cells^44^, which may account for the separation of pig breed in the poly I:C-treated group.

The host’s gene expression programs especially those linked to host defense genes undergo massive changes during pathogenic infections^40^. The epigenetic modulations such as histone modification and DNA methylation can be manipulated by pathogens to influence the host’s gene expression programs associated with defense genes^48^. Our results provided evidence that poly I:C stimulation can trigger changes in DNA methylation to alter the expression of genes related to immune responses. Previous reports have elucidated the potential underlying biology for preferentially inducing epigenetic changes in immune-related genes during pathogenic infections such as modulation of histone acetylation, accession to histone-interacting transcriptional regulators by mimicking the histone H3K4 sequence and encoding proteins that can interact with cellular DNA methyltransferases^49–51^. In addition, DNA methylation is considered as only one of the mechanisms for governing gene expression, and recent studies have documented a not necessary correlation between DNA methylation changes and gene expression variations^15, 46^. The other regulators such as transcription factors are also involved in the regulation of gene expression, which may in part account for the small number of differentially methylated and expressed genes identified in our study.

Functions of DNA methylation vary with the distribution of methylation across the genomic elements. Here we found an overall inverse relationship between promoter methylation and gene expression levels. Among the genes showing differential promoter methylation, several genes such as *TNFRSF9* displayed negative relationships between promoter methylation and gene expression, which may be associated with the transcriptional repression of DNA methylation by blocking the binding of transcriptional activators or recruiting co-repressors^52^. However, a subset of genes exhibited differently from this pattern, which indicated that the changes in promoter methylation and gene expression do not seem to follow a simple logic in PBMCs in response to poly I:C stimulation. Emerging evidence has revealed that the classical epigenomic models that DNA methylation negatively regulates gene expression apply only to a portion of genes. No correlation and positive correlation between promoter methylation and gene expression have been identified, such as in mouse intestinal adenoma and human PBMCs^15, 45^. In addition, DNA methylation is prevalent within gene bodies. It has been shown that gene body methylation is involved in alternative splicing^53^, phenotypic plasticity^54^, and repression of spurious initiation of transcription^55^. Differential DNA methylation in gene bodies was found to be positively correlated with the expression of some genes including the *EBI3* gene, which is concordant with previous reports^56, 57^. The molecular mechanisms may be that the methylation in gene body may promote transcriptional activity through hindering the initiation of intragenic promoters or by influencing repetitive DNAs activities located in the transcriptional units^58^. However, recent studies revealed that not all of the gene body methylation was positively correlated with expression^59^. In this study, we also observed a subset of genes showing positive or negative associations between gene body methylation and expression. A possible rationale is that the function of gene body DNA methylation in gene expression may vary with the targeted genomic elements in gene bodies, such as repetitive elements, enhancer, and alternative promoters. Therefore, more research is required to definitively characterize the function of DNA methylation in genomic features in regulating gene expression.

In summary, we pioneered a comprehensive methylome analysis of pig PBMCs in response to poly I:C stimulation using MeDIP-seq. This study demonstrated clearly methylomic differences after stimulation with poly I:C, and provided an epigenetic overview of this physiological system in response to viral infection. Our findings highlighted the significance of epigenetic modifications that may trigger transcriptional changes in immune-related genes involved in responses of PBMCs to viral infection. Given the variable and complex processes of PBMCs responses to pathogenic infections, further endeavors will be pursued to analyze the genome-wide methylation kinetics of PBMCs at a variety of time points after infections in larger independent cohorts in future studies.

## Materials and Methods

### Sample collection

To increase the biological diversity of the samples and identify the strongest and common signatures of methylation and gene expression, six clinically healthy pigs at 35 days old from two different breeds (Dapulian and Landrace) were used in this study (Table S1). All animals were raised under the same standard indoor conditions, and did not receive any vaccinations except classical swine fever vaccine at day 21 after birth. For each piglet, 20 mL of whole peripheral blood was collected via venipuncture for PBMC separation. All protocols for samples collection of experimental individuals were reviewed and approved by the Institutional Animal Care and Use Committee (IACUC) at China Agricultural University, and all methods were performed in accordance with relevant guidelines and regulations.

### PBMC preparation and poly I:C stimulation

PBMCs were separated from whole peripheral blood by a density gradient centrifugation method using Ficoll-Paque PLUS (GE healthcare). Freshly isolated PBMCs were maintained in RPMI 1640 medium (Hyclone, Logan, UT, USA) supplemented with 10% heat-inactivated fetal calf serum, and the cell viability was confirmed by trypan blue exclusion assays. The PBMC samples obtained from each piglet were classified into two groups that treated with poly I:C at a final concentration of 20 μg/mL or with vehicle (phosphate-buffered saline [PBS]). PBMC cells were maintained in culture for 24 hours^60, 61^ at 37 °C and 5% CO_2_, and then were collected for nucleic acid extraction. Genomic DNA and total RNA were extracted using Qiagen DNeasy Tissue kit (Qiagen, Hilden, Germany) and RNAiso Plus (Takara Biotechnology Co., Ltd, Dalian, China), respectively, and frozen at −80 °C until DNA methylation and RNA-seq analysis.

### MeDIP-seq library preparation and sequencing

We herein used MeDIP-seq to profile the methylomic landscape of PBMC samples derived from poly I:C-treated and control groups. To prepare for MeDIP-seq, 6–10 ug genomic DNA was sheared into random fragments ranging in size from 150 to 800 bp with the AIR™ DNA Fragmentation Kit (Bioo Scientific Corporation). Libraries were prepared according to the Illumina Paired-End protocol, (*i*) end repaire, (*ii*) phosphorylation of the 5′ prime ends, (*iii)* A-tailing of the 3′ ends, *(iv*) ligation of adapters, and were performed with the Paired-End DNA Sample Kit (Illumina, San Diego, USA) following the manufacturer’s guidelines. Adaptor-ligated DNA was immunoprecipitated by highly specific anti-5-methylcytosine monoclonal antibody supplied in the Methylated DNA IP Kit (Zymo Research, Orange, CA, USA). MeDIP efficiency was confirmed by quantitative PCR using SYBR green mastermix (Applied Biosystems, CA, USA), using primers for positive and negative internal control DNA of non-human samples contained in the Magnetic Methylated DNA Immunoprecipitation Kit (Diagenode, Liege, Belgium). The methylated fraction and 10% input DNA were purified with ZYMO DNA Clean & Concentrator-5 columns according to the manufacturer’s instructions and were amplified by adaptor-mediated PCR. Amplification quality and quantity were analyzed by Agilent 2100 Analyzer. Ultra-high-throughput 100 bp paired-end sequencing was performed using the Illumina HiSeq. 2000 following manufacturer’s protocols.

### Quality control and alignment of sequencing reads

Raw sequencing reads were firstly filtered using IlluQC.pl provided in the NGS QC Tookit^62^. For paired end library, only both of the paired reads without primer/adaptor sequences (allowing one bp mismatch) and having at least 70% of bases with PHRED quality score > = 10 were retained. Then we aligned the remaining reads to the pig reference genome (Sscrofa10.2) with Bowtie 2^63^. The pig reference genome was downloaded from the University of California Santa Cruz Genome Bioinformatics Site (UCSC, http://hgdownload.soe.ucsc.edu/goldenPath/susScr3/bigZips/). Finally, we utilized the Samtools^64^ to convert the SAM format file to BAM format and discarded the reads with MAPQ score < 10. To avoid sex-based methylation differences, we removed methylation data for the X and Y chromosomes. The final BAM format files were prepared for subsequent downstream analysis.

### Differential methylation analyses

For differential methylation analyses, after filtering the low quality reads, the MeDIP-seq data were aligned to the pig reference genome (Sscrofa10.2) using Bowtie 2^63^. Read counts for non-overlapping genomic windows of 1 kb were calculated using the MEDIPS package^26^. For each window, the unique read pairs (all reads which aligned to exactly the same start and end positions were replaced by only one representative) that overlap with the window of each sample were counted. The MEDIPS.couplingVector function was used to control for local CpG density for an estimate of actual DNA methylation level. Raw read counts were normalized using the estimateSizeFactors function in the DESeq package in R statistical program^65^. For each window, we carried out paired *t* test on the differences between poly I:C-treated and control groups using the normalized read counts. The resulting *t* statistic can be used to determine the significance^66^. Using the distribution of *t* statistic obtained from each window, we defined a threshold (|t| = 7) for determining DMRs. The windows with the absolute value of *t* statistic that was larger than 7 were taken as DMRs. The improved permutation method was utilized to evaluate the FDR in DMR calling^67^. We randomly shuffled the normalized read counts for each non-DMR window and repeated DMR analysis (10,000 permutations). This analysis indicated that the DMRs were identified with an FDR < 0.1 across the genome.

### Validation of MeDIP-seq data by bisulfite sequencing PCR

Bisulfite sequencing PCR primers were designed by the online MethPrimer program^68^, which are listed in Table S12. Bisulfite treatment of genomic DNA was performed using the EZ DNA Methylation-Gold^™^ Kit (Zymo Research, D5006). PCR reactions were then undertaken using the ZymoTaq^™^ PreMix (Zymo Research, E2004) following the manufacturer’s protocols. The PCR products were purified and cloned into the pMD18-T vector (TaKaRa biotechnology Co., Ltd, Dalian, China). More than ten positive clones for each subject were randomly selected for sequencing. The final sequences were analyzed by the online software QUMA^69^.

### RNA-seq library preparation, sequencing and data analysis

A total of 10 ug total RNA was treated with poly-T oligo attached magnetic beads to isolate Poly (A) mRNA. The captured mRNA was fragmented randomly into smaller pieces using RNA fragmentation buffer (NEB, Beijing, China) following the manufacturer’s procedure. Then the cleaved RNA fragments were reverse-transcribed to construct the final cDNA library according to the protocols for the mRNA-Seq sample preparation kit (Illumina, San Diego, USA), and the average insert size for the paired-end libraries was 300 bp ( ± 50 bp). After that, 100 bp paired-end sequencing was performed on the Illumina Hiseq. 2000 following the vendor’s recommended protocols.

We filtered out low quality RNA-seq reads that contain sequencing adaptor or sequencing primer or with base quality score lower than 20. The clean reads were then aligned to the pig reference genome using TopHat2^70^. The script htseq-count included in HTSeq was used to count for each gene how many aligned reads overlapped its exons. The edgeR^71^ software was used for read counts normalization and differentially expressed gene determination using the procedure of a generalization of paired t-test for paired samples. We defined a threshold of FDR < 0.05 for determining the significance of expressed genes.

### Quantitative real-time PCR

Total RNA was purified and reversely transcribed into cDNA using PrimerScript^®^ RT reagent Kit with gDNA Eraser (Takara Biotechnology (Dalian) Co., Ltd) following the manufacturer’s instructions. Quantities of mRNA were then measured with qRT-PCR using a LightCycler^®^ 480 Real-Time PCR System (Roche, Hercules, CA, USA). The qRT-PCR assays were performed with a volume of 20 μL containing 10 μL SYBR Green Mixture, 7 μL deionized water, 1 μL template of cDNA, 1 μL of each primer. The thermal conditions were as follows: 95 °C for 5 min, 45 cycles of 95 °C for 10 sec, 60 °C for 10 sec, 72 °C for 10 sec. Primer sequences used for qRT-PCR assays are presented in Table S13. Each qPCR assay was carried out in triplicate. The relative gene expression was calculated by using the 2^−*ΔΔ*Ct^ method^72^.

### Functional enrichment and network analysis

The DAVID (Database for Annotation, Visualization and Integrated Discovery) web server (http://david.abcc.ncifcrf.gov/) was used to investigate the biological relevance of the differentially methylated genes. Since only a limited number of genes in the pig genome have been annotated, the identified genes were firstly mapped to their corresponding human orthologs, and then the gene lists were submitted into the databases for functional enrichment analysis. Annotation terms with a *P* value less than 0.05 were considered to be statistically significant.

### Annotation of DMRs

DMRs were annotated with Ensembl genes, transcripts, promoters, CpG islands (CGIs) and repeatmask. We obtained the genomic locations of these genomic features from the UCSC pig reference genome (Sscrofa10.2). As described in previous study^2^, promoters were defined as −2,200 bp to +500 bp of the transcription start site. The promoters were further classified into three groups based on the CpG representation as previously described^37^, i.e., low CpG (LCP), intermediate CpG (ICP) and high CpG content promoters (HCP). DNA regions greater than 200 bp with a C + G content equal to or greater than 50% and an observed CpG/expected CpG in excess of 0.6 were defined as CGIs^73^. The sequences up to 2 kb distant from CpG islands were termed as CpG island shores^74^.

## Electronic supplementary material

Additional file 1

Additional file 2

## References

[1] Jaenisch R, Bird A (2003). Epigenetic regulation of gene expression: how the genome integrates intrinsic and environmental signals. Nat Genet.

[2] Koga Y (2009). Genome-wide screen of promoter methylation identifies novel markers in melanoma. Genome Res.

[3] Feinberg AP (2007). Phenotypic plasticity and the epigenetics of human disease. Nature.

[4] Charlton J (2014). Methylome analysis identifies a Wilms tumor epigenetic biomarker detectable in blood. Genome Biol.

[5] Davies MN (2014). Hypermethylation in the ZBTB20 gene is associated with major depressive disorder. Genome Biol.

[6] Wiencke JK (2016). The DNA methylation profile of activated human natural killer cells. Epigenetics.

[7] Schoenborn JR (2007). Comprehensive epigenetic profiling identifies multiple distal regulatory elements directing transcription of the gene encoding interferon-γ. Nat Immunol.

[8] Northrop JK, Thomas RM, Wells AD, Shen H (2006). Epigenetic remodeling of the IL-2 and IFN-γ loci in memory CD8 T cells is influenced by CD4 T cells. J Immunol.

[9] Steinfelder S (2011). Epigenetic modification of the human CCR6 gene is associated with stable CCR6 expression in T cells. Blood.

[10] Yamashita M (2008). Bmi1 regulates memory CD4 T cell survival via repression of the Noxa gene. J Exp Med.

[11] Youngblood B (2011). Chronic virus infection enforces demethylation of the locus that encodes PD-1 in antigen-specific CD8+ T cells. Immunity.

[12] Li Y (2010). The DNA methylome of human peripheral blood mononuclear cells. PLoS Biol.

[13] Matei D (2012). Epigenetic resensitization to platinum in ovarian cancer. Cancer Res.

[14] Di Francesco A (2015). Global changes in DNA methylation in Alzheimer’s disease peripheral blood mononuclear cells. Brain Behav Immun.

[15] Lam LL (2012). Factors underlying variable DNA methylation in a human community cohort. Proc Natl Acad Sci USA.

[16] Kawai T, Akira S (2006). Innate immune recognition of viral infection. Nat Immunol.

[17] Li M (2012). An atlas of DNA methylomes in porcine adiposeand muscle tissues. Nat Commun.

[18] Choi M (2015). Genome-wide analysis of DNA methylation in pigs using reduced representation bisulfite sequencing. DNA Res.

[19] Qu X (2015). Sulforaphane epigenetically regulates innate immune responses of porcine monocyte-derived dendritic cells induced with lipopolysaccharide. PLoS one.

[20] Adler M, Murani E, Brunner R, Ponsuksili S, Wimmers K (2013). Transcriptomic response of porcine PBMCs to vaccination with tetanus toxoid as a model antigen. PLoS one.

[21] Gao Y (2010). Transcriptome analysis of porcine PBMCs after *in vitro* stimulation by LPS or PMA/ionomycin using an expression array targeting the pig immune response. BMC Genomics.

[22] Zhuge ZY (2012). Effects of astragalus polysaccharide on immune responses of porcine PBMC stimulated with PRRSV or CSFV. PLoS one.

[23] Meurens F, Summerfield A, Nauwynck H, Saif L, Gerdts V (2012). The pig: a model for human infectious diseases. Trends Microbiol.

[24] Walters EM (2012). Completion of the swine genome will simplify the production of swine as a large animal biomedical model. BMC Med Genomics.

[25] Fernandez-Morera JL, Calvanese V, Rodriguez-Rodero S, Menendez-Torre E, Fraga MF (2010). Epigenetic regulation of the immune system in health and disease. Tissue Antigens.

[26] Lienhard M, Grimm C, Morkel M, Herwig R, Chavez L (2014). MEDIPS: genome-wide differential coverage analysis of sequencing data derived from DNA enrichment experiments. Bioinformatics.

[27] Blasco MA (2007). The epigenetic regulation of mammalian telomeres. Nat Rev Genet.

[28] Gonzalo S (2006). Dna methyltransferases control telomere length and telomere recombination in mammalian cells. Nat Cell Biol.

[29] Lister R (2009). Human dna methylomes at base resolution show widespread epigenomic differences. Nature.

[30] Huang YZ (2014). Genome-wide dna methylation profiles and their relationships with mrna and the microrna transcriptome in bovine muscle tissue (bos taurine). Sci Rep.

[31] Li Q (2011). Genome-wide mapping of dna methylation in chicken. PLoS One.

[32] Jones PA (2012). Functions of DNA methylation: islands, start sites, gene bodies and beyond. Nat Rev Genet.

[33] Tarakhovsky A (2010). Tools and landscapes of epigenetics. Nat Immunol.

[34] Eckhardt F (2006). Dna methylation profiling of human chromosomes 6, 20 and 22. Nat Genet.

[35] Schneider M (2012). The innate immune sensor nlrc3 attenuates toll-like receptor signaling via modification of the signaling adaptor traf6 and transcription factor nf-κb. Nat Immunol.

[36] Hu H (2006). Foxp1 is an essential transcriptional regulator of B cell development. Nat Immunol.

[37] Nestor CE (2014). DNA methylation changes separate allergic patients from healthy controls and may reflect altered cd4 t-cell population structure. PLoS Genet.

[38] Weber M (2007). Distribution, silencing potential and evolutionary impact of promoter dna methylation in the human genome. Nat Genet.

[39] Huang CC (2006). A pathway analysis of poly(i:c)-induced global gene expression change in human peripheral blood mononuclear cells. Physiol Genomics.

[40] Boldrick JC (2002). Stereotyped and specific gene expression programs in human innate immune responses to bacteria. Proc Natl Acad Sci USA.

[41] Croft M (2009). The role of TNF superfamily members in T-cell function and diseases. Nat Rev Immunol.

[42] Lepiller Q (2015). Antiviral and immunoregulatory effects of indoleamine-2, 3-dioxygenase in hepatitis C virus infection. J Innate Immune.

[43] Shinsuke N, Hiroshi I (2013). Overexpression of Epstein–Barr virus-induced gene 3 protein (EBI3) in MRL/lpr mice suppresses their lupus nephritis by activating regulatory T cells. Autoimmunity.

[44] Obata Y, Furusawa Y, Hase K (2015). Epigenetic modifications of the immune system in health and disease. Immunol Cell Biol.

[45] Feber A (2011). Comparative methylome analysis of benign and malignant peripheral nerve sheath tumors. Genome Res.

[46] Grimm C (2013). DNA–methylome analysis of mouse intestinal adenoma identifies a tumour-specific signature that is partly conserved in human colon cancer. PLoS Genet.

[47] Xing (2014). Genome-wide gene expression profiles in lung tissues of pig breeds differing in resistance to porcine reproductive and respiratory syndrome virus. PLoS One.

[48] Paschos K, Allday MJ (2010). Epigenetic reprogramming of host genes in viral and microbial pathogenesis. Trends Microbiol.

[49] Garcia-Garcia JC, Barat NC, Trembley SJ, Dumler JS (2009). Epigenetic silencing of host cell defense genes enhances intracellular survival of the rickettsial pathogen Anaplasma phagocytophilum. PLoS Pathog.

[50] Marazzi I (2012). Suppression of the antiviral response by an influenza histone mimic. Nature.

[51] Shamay M, Krithivas A, Zhang J, Hayward SD (2006). Recruitment of the de novo DNA methyltransferase Dnmt3a by Kaposi’s sarcoma-associated herpesvirus LANA. Proc Natl Acad Sci USA.

[52] Klose RJ, Bird AP (2006). Genomic DNA methylation: the mark and its mediators. Trends Biochem Sci.

[53] Foret S (2012). DNA methylation dynamics, metabolic fluxes, gene splicing, and alternative phenotypes in honey bees. Proc Natl Acad Sci USA.

[54] Kucharski R, Maleszka J, Foret S, Maleszka R (2008). Nutritional control of reproductive status in honeybees via DNA methylation. Science.

[55] Huh I, Zeng J, Park T, Yi SV (2013). DNA methylation and transcriptional noise. Epigenetics & Chromatin.

[56] Yang X (2014). Gene body methylation can alter gene expression and is a therapeutic target in cancer. Cancer Cell.

[57] Varley KE (2013). Dynamic DNA methylation across diverse human cell lines and tissues. Genome Res.

[58] Maunakea AK (2010). Conserved role of intragenic DNA methylation in regulating alternative promoters. Nature.

[59] Kulis M (2012). Epigenomic analysis detects widespread gene-body DNA hypomethylation in chronic lymphocytic leukemia. Nat Genet.

[60] Alexopoulou L, Holt AC, Medzhitov R, Flavell RA (2001). Recognition of double-stranded RNA and activation of NF-κB by Toll-like receptor 3. Nature.

[61] Gitlin L (2006). Essential role of mda-5 in type I IFN responses to polyriboinosinic: polyribocytidylic acid and encephalomyocarditis picornavirus. Proc Natl Acad Sci USA.

[62] Patel RK, Jain M (2012). NGS QC Toolkit: a toolkit for quality control of next generation sequencing data. PLoS One.

[63] Langmead B, Salzberg SL (2012). Fast gapped-read alignment with Bowtie 2. Nat Methods.

[64] Li H (2009). The sequence alignment/map format and SAMtools. Bioinformatics.

[65] Anders S, Huber W (2010). Differential expression analysis for sequence count data. Genome Biol.

[66] Cui X, Churchill GA (2003). Statistical tests for differential expression in cDNA microarray experiments. Genome Biol.

[67] Xie Y, Pan W, Khodursky AB (2005). A note on using permutation-based false discovery rate estimates to compare different analysis methods for microarray data. Bioinformatics.

[68] Li LC, Dahiya R (2002). MethPrimer: designing primers for methylation PCRs. Bioinformatics.

[69] Kumaki Y, Oda M, Okano M (2008). QUMA: quantification tool for methylation analysis. Nucleic Acids Res.

[70] Kim D (2013). TopHat2: accurate alignment of transcriptomes in the presence of insertions, deletions and gene fusions. Genome Biol.

[71] Robinson MD, McCarthy DJ, Smyth G (2010). K. edgeR: a Bioconductor package for differential expression analysis of digital gene expression data. Bioinformatics.

[72] Livak KJ, Schmittgen TD (2001). Analysis of relative gene expression data using real-time quantitative PCR and the 2(T)(−Delta Delta C) method. Methods.

[73] Gardiner-Garden M, Frommer M (1987). Cpg Islands in vertebrate genomes. J Mol Biol.

[74] Irizarry RA (2009). The human colon cancer methylome shows similar hypo-and hypermethylation at conserved tissue-specific CpG island shores. Nat Genet.

