# Supplementary material for: Genome-wide DNA methylation and transcriptome analyses reveal genes involved in immune responses of pig peripheral blood mononuclear cells to poly I:C
Source: Sci Rep. 2017 Aug 29;7:9709. doi: 10.1038/s41598-017-10648-9 (PMC5575306; doi:10.1038/s41598-017-10648-9)
Supplement: Supplementary file 2 — Additional file 2 [file 41598_2017_10648_MOESM2_ESM.doc]

**Genome-wide DNA methylation and transcriptome analyses reveal genes involved in immune responses of pig peripheral blood mononuclear cells to poly I:C**

Haifei Wang^1,†^, Jiying Wang^1,2,†^, Chao Ning^1,†^, Xianrui Zheng^1^, Jinlian Fu^1^, Aiguo Wang^1^, Qin Zhang^1^, Jian-Feng Liu^1,*^

^1^Key Laboratory of Animal Genetics, Breeding and Reproduction, Ministry of Agriculture, College of Animal Science and Technology, China Agricultural University, Beijing 100193, China

^2^Shandong Key Laboratory of Animal Disease Control and Breeding, Institute of Animal Science and Veterinary Medicine, Shandong Academy of Agricultural Sciences, Jinan 250100, China

^*^ Corresponding author, Email: liujf@cau.edu.cn

^†^These authors contributed equally to this work.

**Supplementary figures**


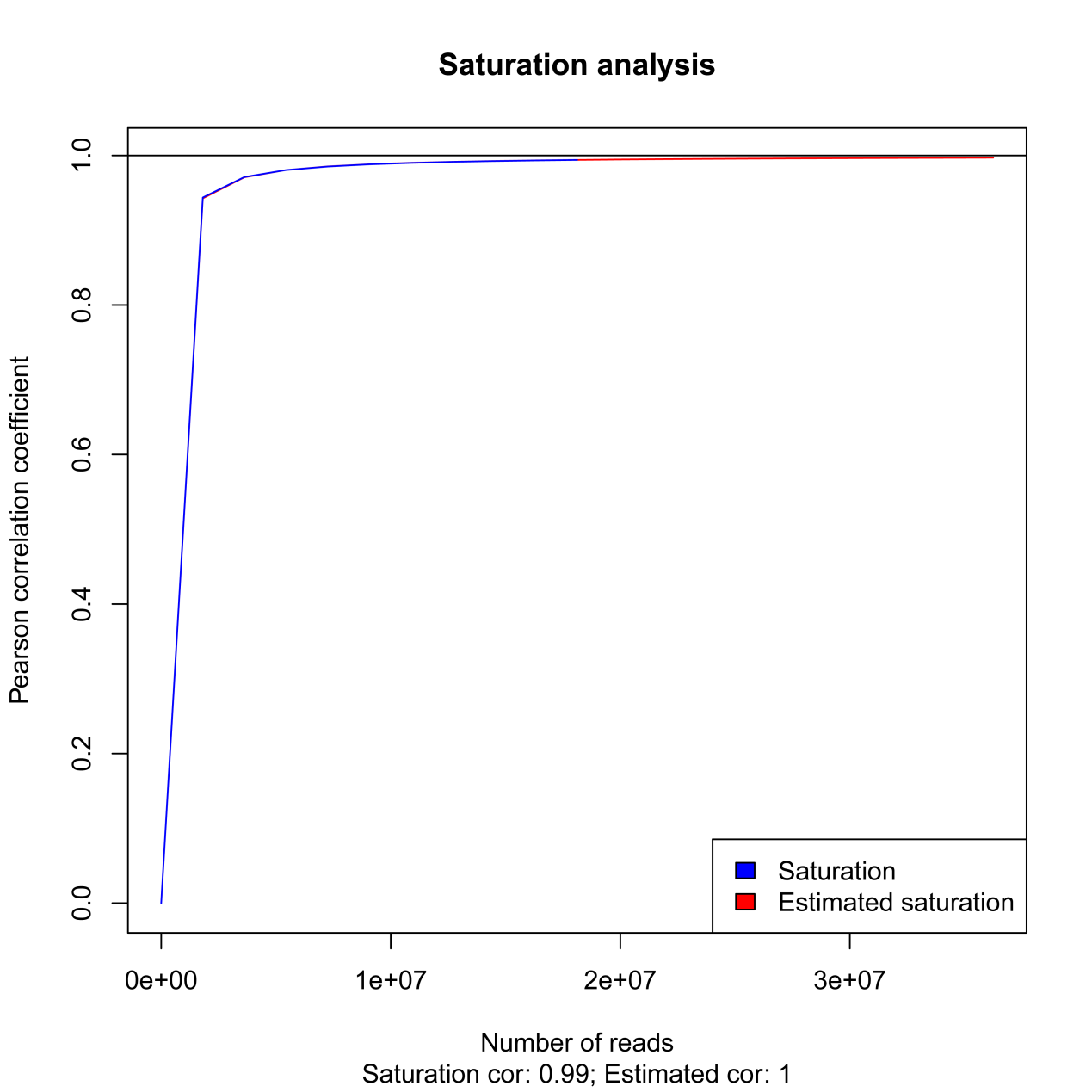


**Figure S1.** Saturation analysis of MeDIP-seq data. Saturation analysis for MeDIP-seq data of each sample was done using the MEDIPS package. Shown is an example of saturation analysis results derived from one randomly selected sample.


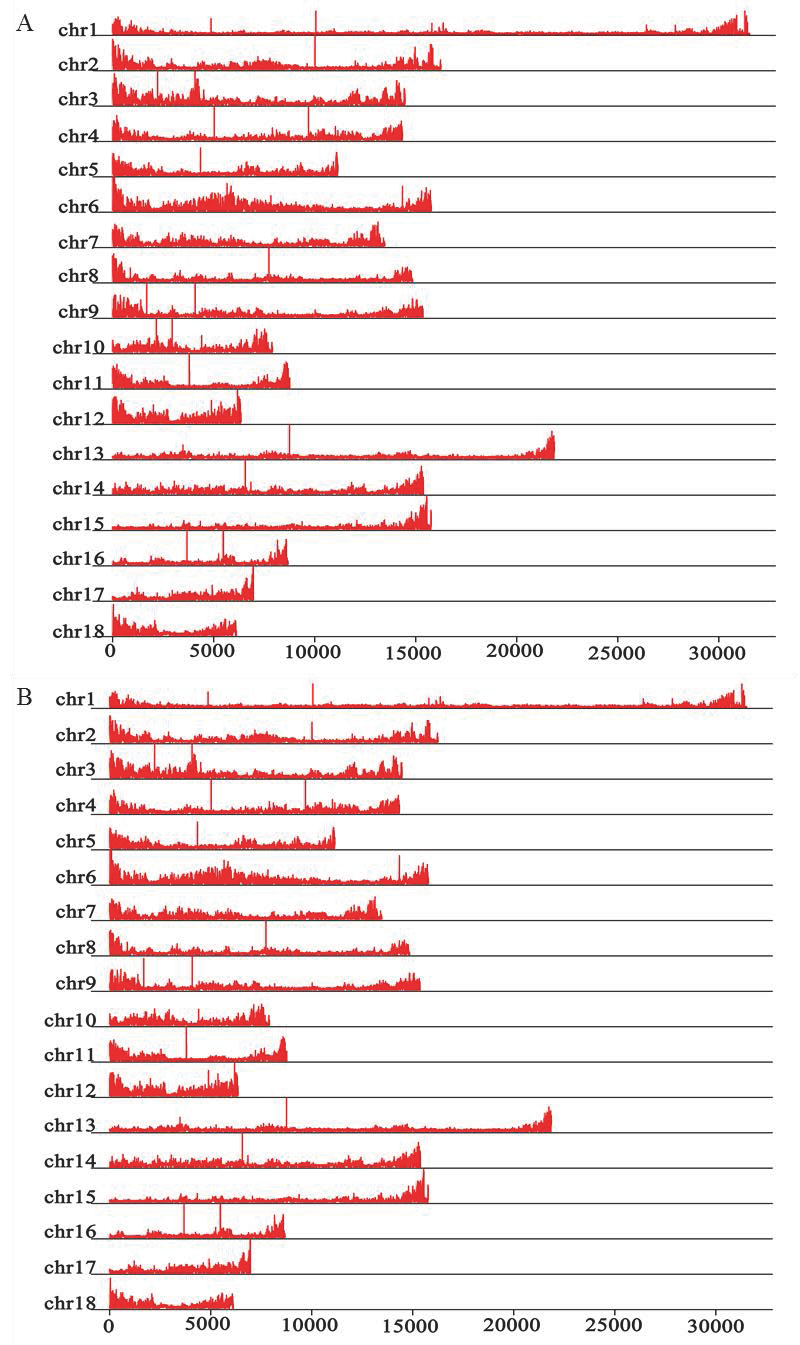


**Figure S2.** Distribution of MeDIP-seq reads on each chromosome in the two groups. We measured the methylation levels on each chromosome using a 10 kb sliding window. The x axis indicates the number of windows and the y axis indicates the normalized read counts of each window. The poly I:C-treated group (A) and the control group (B).


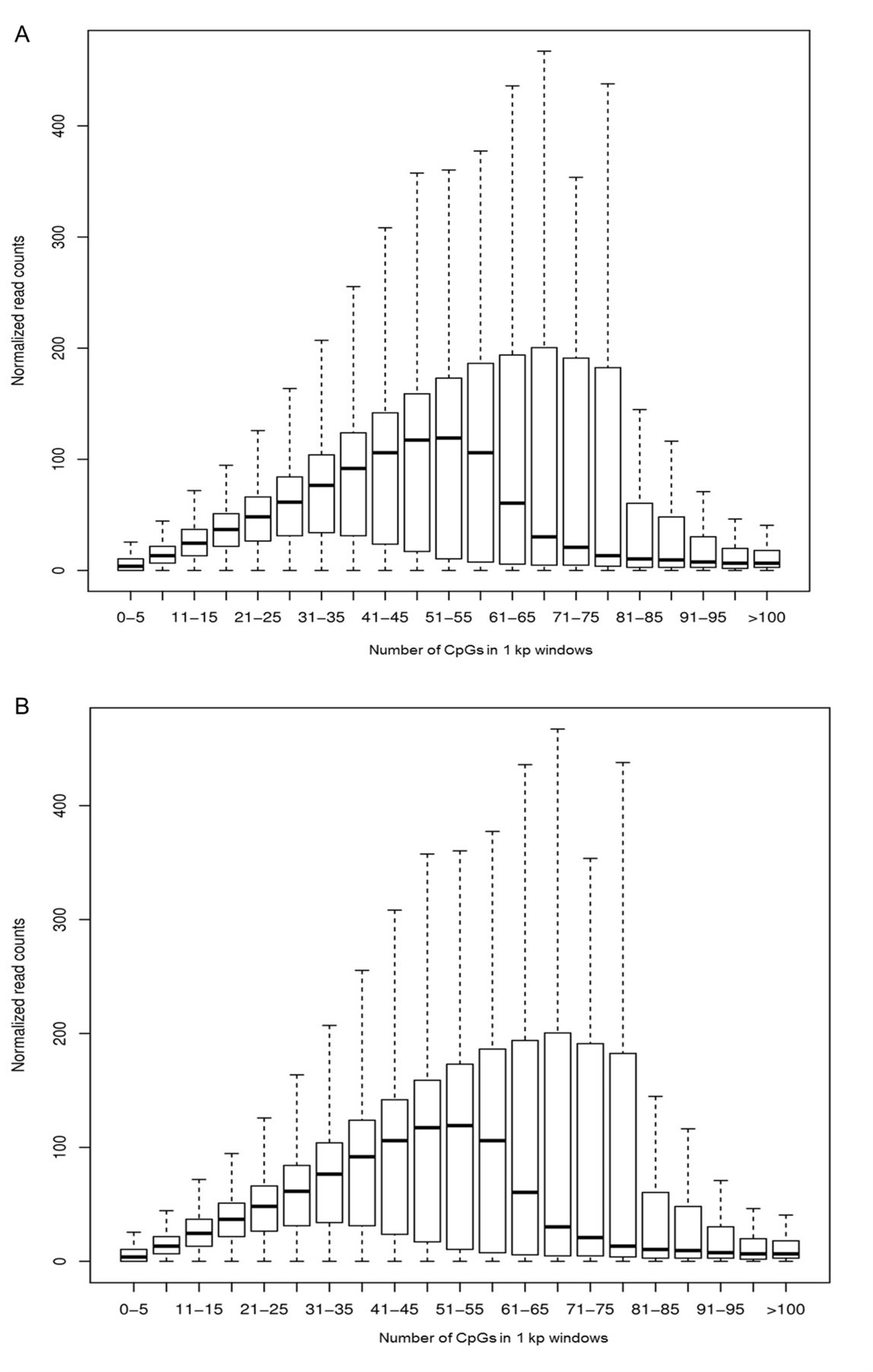


**Figure S3.** Distribution of sequencing reads against the CpG density in the poly I:C-treated group (A) and untreated group (B). The number of CpGs in 1 kp reflects the CpG density of a specific region. Boxes indicate the interquartile range between the first and third quartiles, and the bold line indicates the median. Whiskers denote the [minimum](http://www.iciba.com/minimum) and maximum within 1.5 times the interquartile range from the first and third quartiles.

**
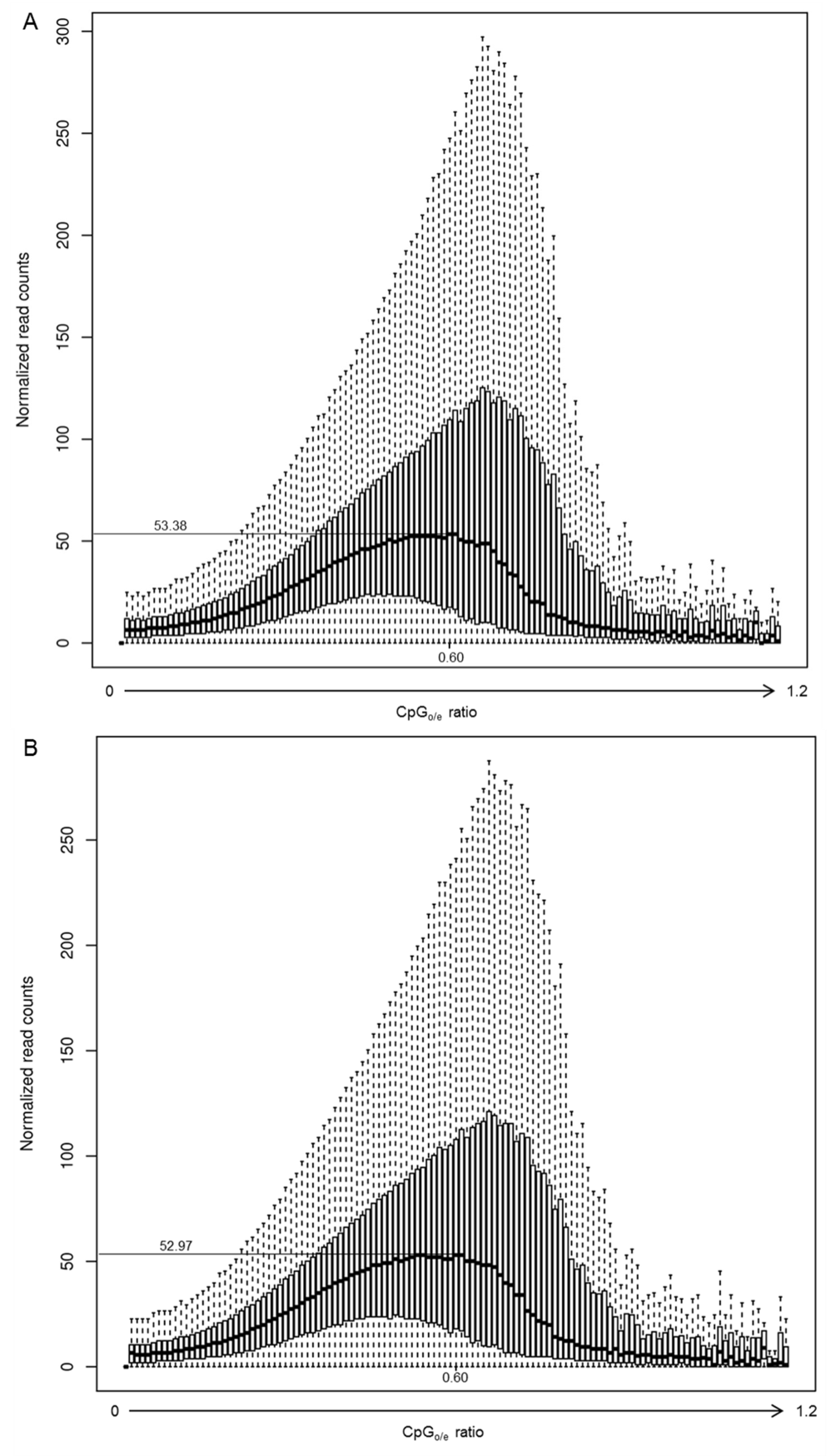
**

**Figure S4.** Distribution of sequencing reads against the CpGo/e ratio. The CpGo/e ratio of each 1 kb window across the pig genome was calculated in poly I:C-treated (A) and untreated group (B). Boxes indicate the interquartile range between the first and third quartiles, and the bold line indicates the median. Whiskers denote the [minimum](http://www.iciba.com/minimum) and maximum within 1.5 times the interquartile range from the first and third quartiles.


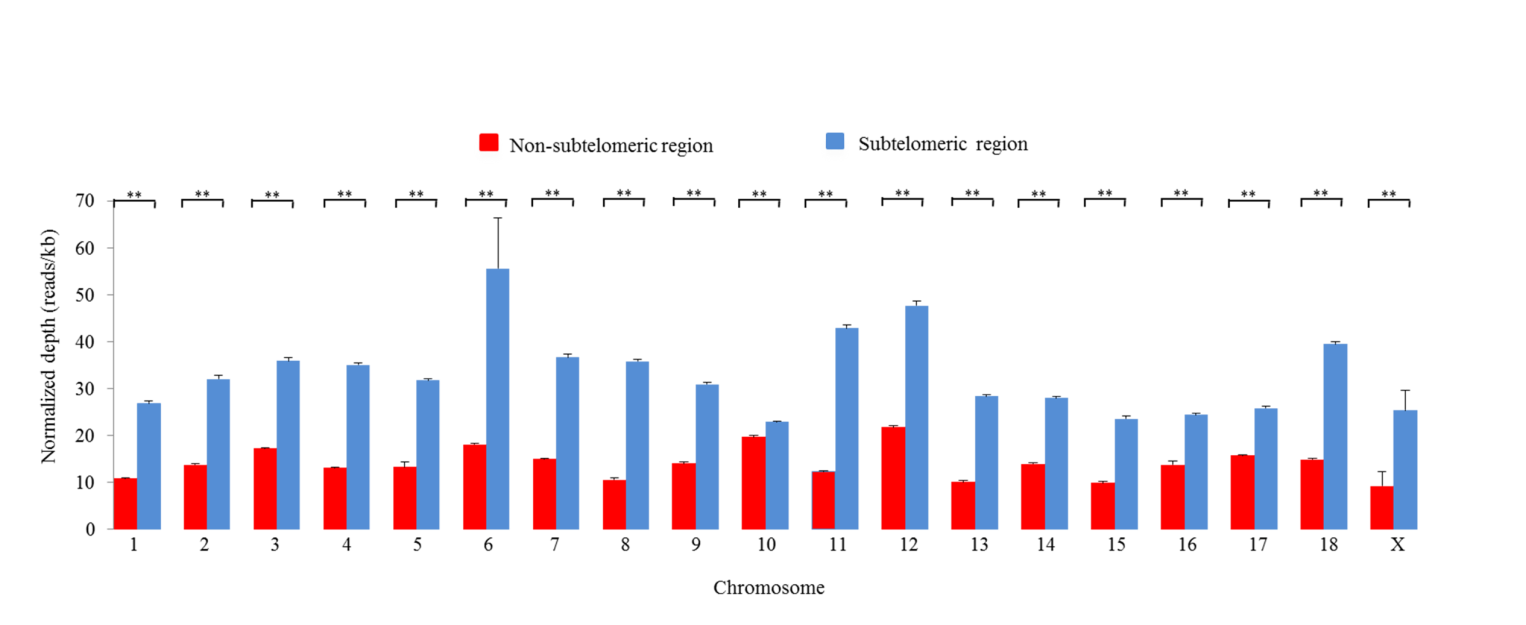


**Figure S5.** DNA methylation levels of the subtelomeric (7 Mb downstream of the telomere) and non-subtelomeric regions in each chromosome. ^**^P indicates a significant difference at the 0.01 significance level (Student's t-test). Bars represent means **±** standard deviation (N = 12).


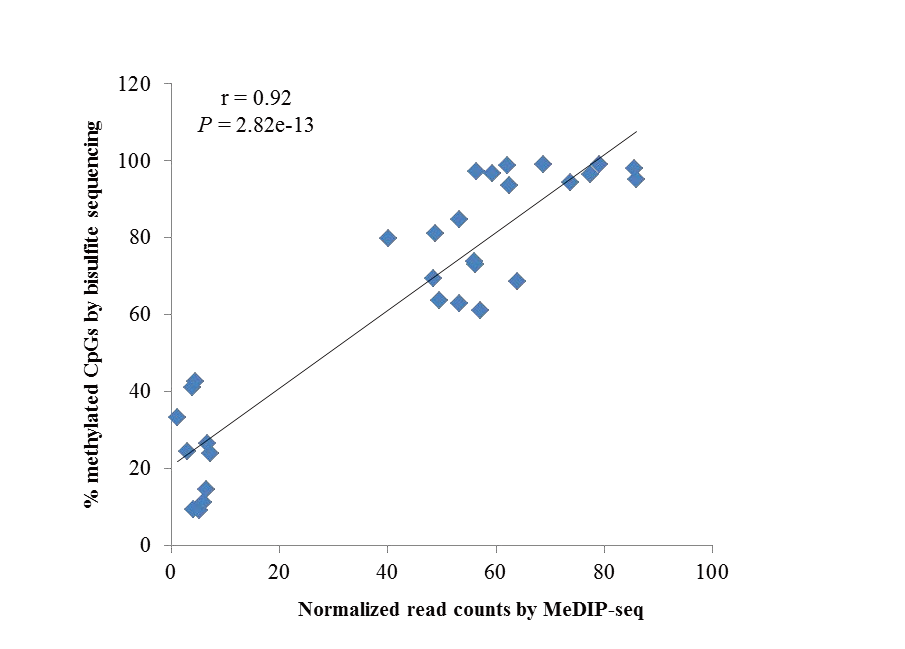


**Figure S6.** Correlation analysis between the MeDIP-seq data and bisulfite sequencing data. Pearson’s correlation coefficient is denoted above the linear regression curve.


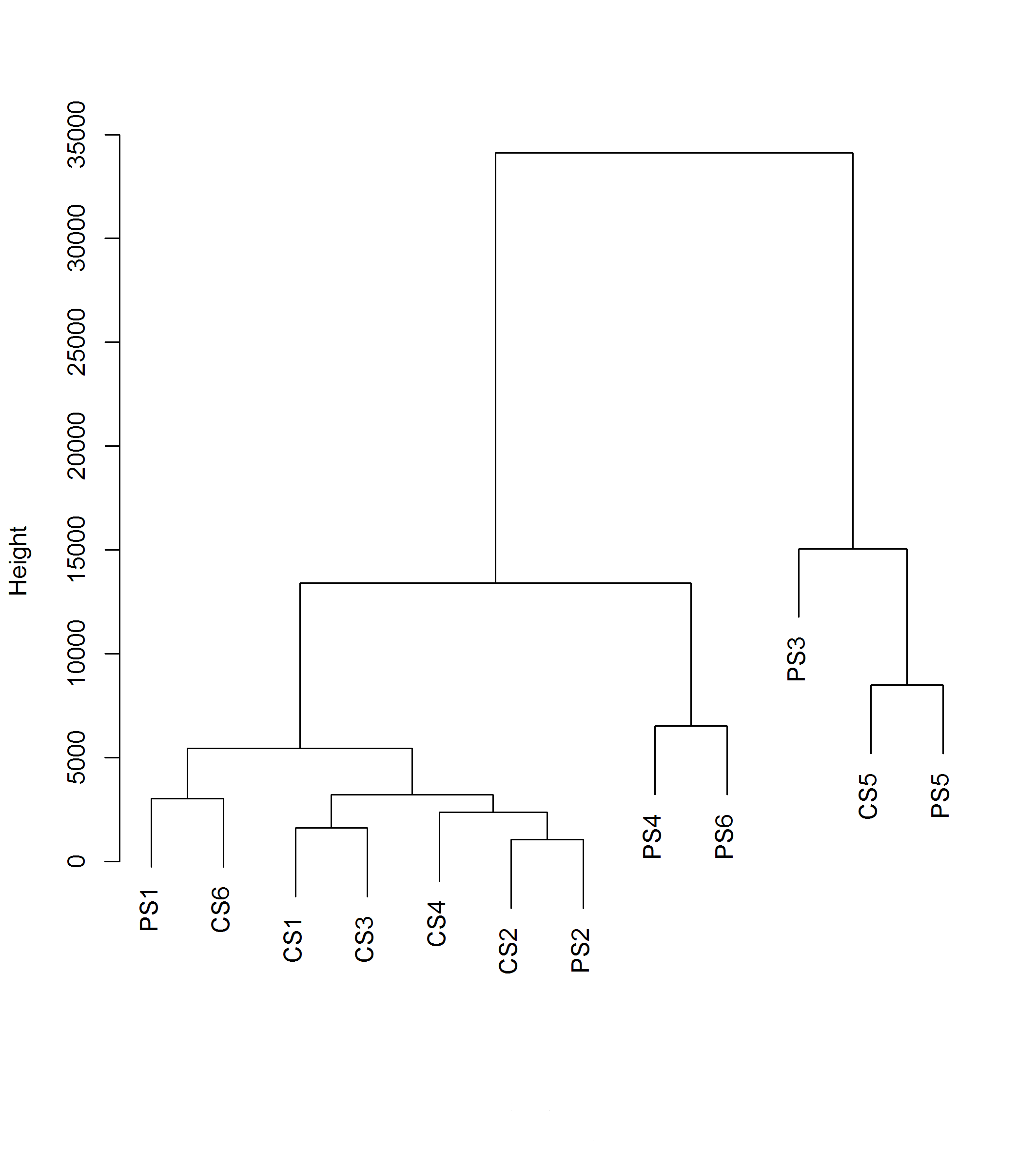


**Figure S7. Unsupervised clustering analysis of samples by mRNA expression profile.** Clustering was performed using the hclust function in R.


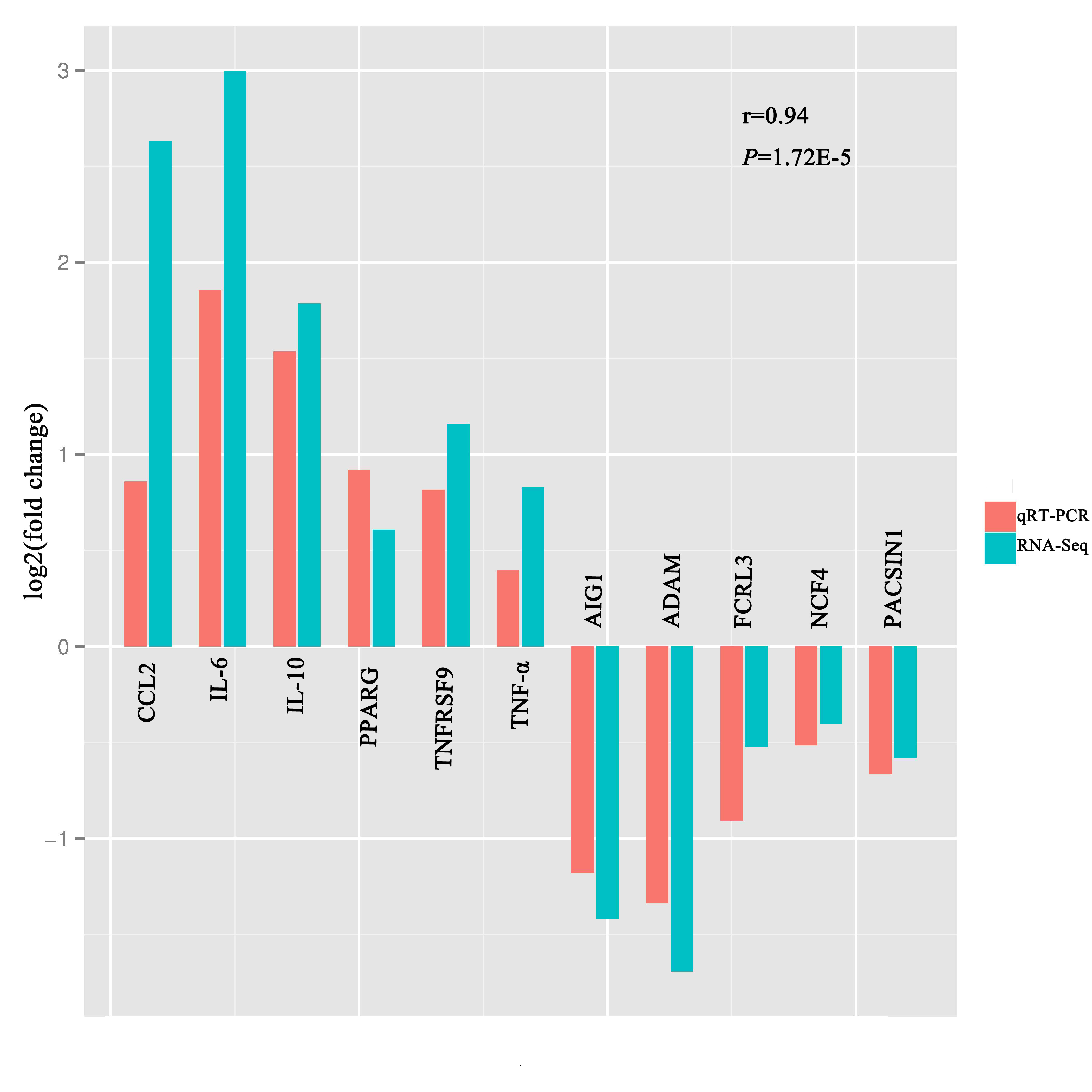
**Figure S8. Comparison of fold changes between RNA-seq analysis results and qRT-PCR data in ten differentially expressed genes.** Fold changes are expressed as ratio of gene expression after poly I:C stimulation to the untreated control. The red and cyan bars represent the RNA-seq and qRT-PCR data, respectively.


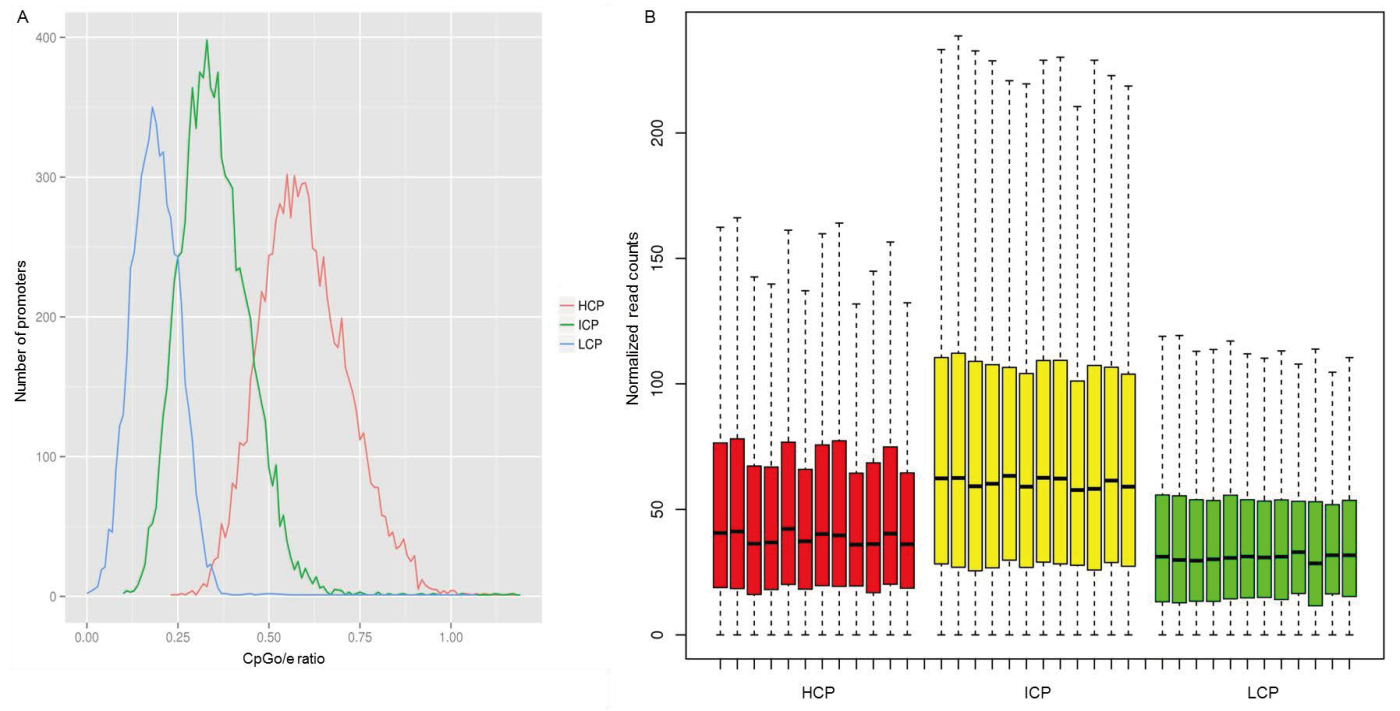


**Figure S9. Methylation levels of three categories of promoters in relation to CpG frequency.** All promoters in the pig genome were classified into three classes (HCP = 8635, ICP = 8599, and LCP = 5577) based on the CpG representation. The DNA methylation level of three categories of promoters in each individual was represented by the normalized read counts. Boxes indicate the interquartile range between the first and third quartiles, and the bold line indicates the median. Whiskers denote the [minimum](http://www.iciba.com/minimum) and maximum within 1.5 times the interquartile range from the first and third quartiles.


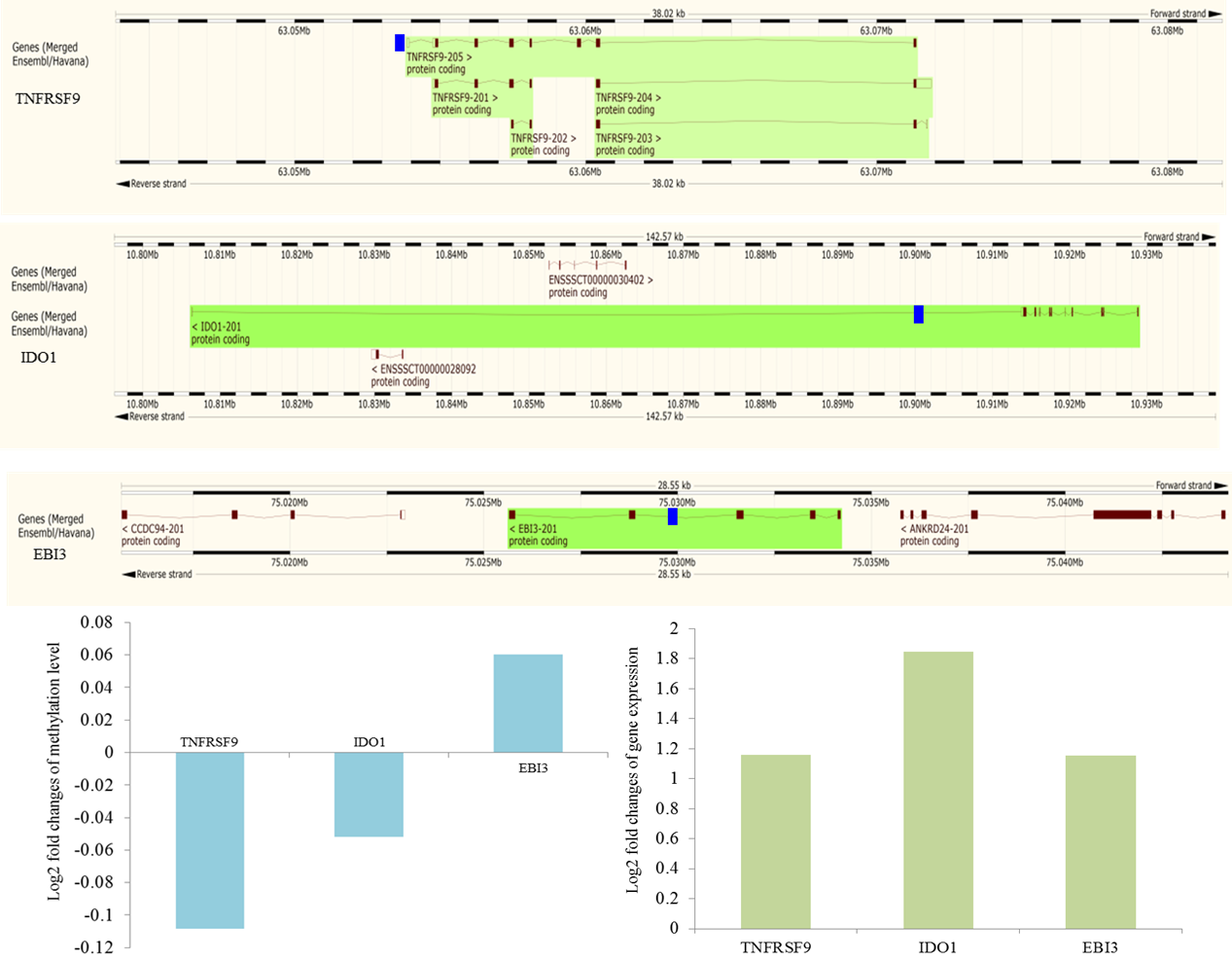


**Figure S10. Genomic locations and degrees of the methylation and expression differences of the *TNFRSF9*, *IDO1* and *EBI3* genes.** The blue boxes in the up panel denote the DMRs (chr6: 63053001-63054000, chr17: 10902001-10903000 and chr2: 75029001-75030000) associated with the three genes, respectively.
